# Machine Learning-Based Approaches for Breast Density Estimation from Mammograms: A Comprehensive Review

**DOI:** 10.3390/jimaging11020038

**Published:** 2025-01-26

**Authors:** Khaldoon Alhusari, Salam Dhou

**Affiliations:** Department of Computer Science and Engineering, American University of Sharjah, Sharjah P.O. Box 26666, United Arab Emirates; sdhou@aus.edu

**Keywords:** breast cancer, breast density, mammographic density estimation, machine learning

## Abstract

Breast cancer, as of 2022, is the most prevalent type of cancer in women. Breast density—a measure of the non-fatty tissue in the breast—is a strong risk factor for breast cancer that can be estimated from mammograms. The importance of studying breast density is twofold. First, high breast density can be a factor in lowering mammogram sensitivity, as dense tissue can mask tumors. Second, higher breast density is associated with an increased risk of breast cancer, making accurate assessments vital. This paper presents a comprehensive review of the mammographic density estimation literature, with an emphasis on machine-learning-based approaches. The approaches reviewed can be classified as visual, software-, machine learning-, and segmentation-based. Machine learning methods can be further broken down into two categories: traditional machine learning and deep learning approaches. The most commonly utilized models are support vector machines (SVMs) and convolutional neural networks (CNNs), with classification accuracies ranging from 76.70% to 98.75%. Major limitations of the current works include subjectivity and cost-inefficiency. Future work can focus on addressing these limitations, potentially through the use of unsupervised segmentation and state-of-the-art deep learning models such as transformers. By addressing the current limitations, future research can pave the way for more reliable breast density estimation methods, ultimately improving early detection and diagnosis.

## 1. Introduction

Breast cancer was the second most common type of cancer in 2022, with over 2.3 million cases recorded in 185 countries [1]. Moreover, in females, it was responsible for 23.8% of all cancer cases and 15.4% of all cancer-related deaths. Given its high prevalence and steep mortality rates, it is undoubtedly a dangerous prospect for women. However, it has been proven that the risks of mortality can be mitigated if breast cancer patients are diagnosed early and consequently treated effectively [2]. The most effective method for early detection is mammography, given its efficacy in detecting small tumors before they grow large enough to cause symptoms [3]. Mammography is an X-ray-based screening procedure that produces images of the breast—referred to as mammograms—which allow for the early detection of tumors, boasting a reported sensitivity of 86.9% [3].

In addition to directly aiding in the early detection of growths, mammograms can be used by radiologists to ascertain a strong risk factor for breast cancer, known as mammographic (or breast) density. Breast density is a measure of the amount of radio-dense fibro-glandular (i.e., non-fatty) tissue within the breast [4]. Women with high breast density have been shown to be far more likely to develop breast cancer, marking breast density as a critical risk factor and one worth studying [5].

**Figure 1:**
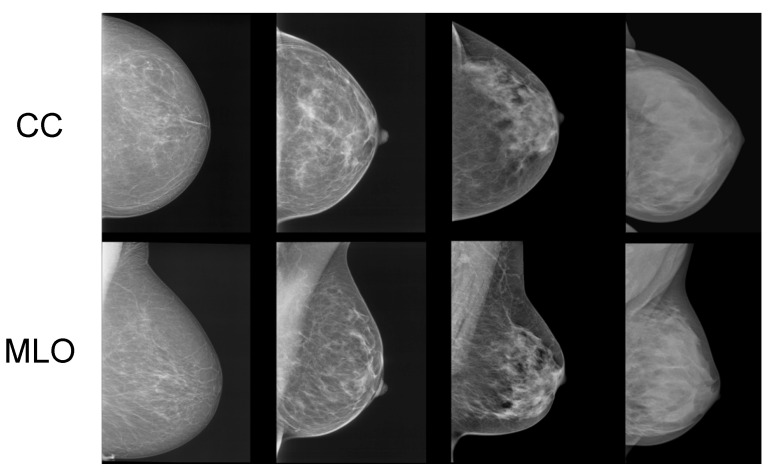
Samples of craniocaudal (CC) and mediolateral oblique (MLO) view mammograms with differing densities [7].

In Figure 1, samples of mammograms with varying densities are provided. Denser tissue appears light within a mammogram, contrasting with the dark appearance of fatty tissue [5]. Potentially harmful growths and tumors also appear light within mammograms. While this contrast makes it easier for radiologists to identify tumors in low-density (fatty) breasts, it notably reduces the sensitivity of mammograms in cases of highly dense breasts [6]. This is because dense tissue can mask potential tumors [6].

The efficacy of mammography relies heavily on the subjective interpretation of the attending radiologist. Though radiologists tend to agree on the majority of cases, there is notable subjectivity in cases of highly dense breasts [8,9]. In ref. [8], 21 expert radiologists classified 100 mammograms into 4 density categories, with an overall agreement of only 61.4%. The experience of the radiologists in this work ranged from 4 to 22 years of clinical experience, with an average of 12 years. Another study, ref. [9], involving the assessments of 83 radiologists found that the percentage of breasts classified as dense varied widely, ranging from 6.3% to 84.5% depending on the radiologist’s interpretation. This amount of subjectivity may dangerously influence a radiologist’s decision as to whether harmful lesions are present within a mammogram. Significant inter-observer variability has also been observed in relation to breast cancer screening in mammograms, as seen in [10], as well as in the measurement of breast cancer proliferation markers, such as Ki-67 [11].

In addition, it is fairly common for cancers to go undetected in mammograms and only be identified later during retrospective reviews [12]. This can often be attributed to misinterpretations of the true breast density, which can result from factors such as the assumption of the asymmetry of fibro-glandular tissue—for example, dense tissue may appear in one mammographic view of the breast but not in the other, leading a radiologist to mistake a lesion for dense tissue. Moreover, errors in evaluating breast density can lead radiologists to make biased decisions, including opting for more invasive procedures. This occurs because mammogram sensitivity decreases significantly for extremely dense breasts, with the research in [6] suggesting it can be as low as 40%. Furthermore, radiologists often miss cancers in mammograms due to cognitive biases, misinterpretations, and other factors discussed in [12]. As such, while radiologist interpretation is generally sound, it is not without its challenges.

Given these issues, it is clear that there is a need for a less subjective, more accurate, and easily integrable method of estimating breast density. Various studies in the literature have tackled the issue of breast density estimation from mammograms. The purpose of this paper is to provide a comprehensive review of the literature surrounding density estimation methods, including visual, software-based, and artificial intelligence (AI)-based approaches. While other surveys exist, they are either outdated [13,14], not primarily focused on automated, AI-based approaches [15,16,17,18,19], or have concentrated on other imaging modalities [20]. Therefore, the necessity for a new review is evident, as it will address these gaps. The emphasis on AI-based approaches is also important given the recent deployment of machine learning for various tasks related to breast imaging, such as tumor detection and classification, mammographic image improvement, and breast cancer risk assessment, as well as breast density estimation [21].

The rest of the paper is organized as follows: In Section 2, the research methodology, as well as the inclusion and exclusion criteria, are discussed. In Section 3, visual, software-based, machine-learning-based, and segmentation-based approaches to breast density estimation are reviewed. In Section 4, the limitations of the current approaches are discussed and future research directions are outlined. Lastly, a conclusion is provided in Section 5.

## 2. Methods

To obtain the papers to be reviewed, several online databases were searched for papers that proposed automated breast density estimation methods. The main search keywords were “breast density”, “mammographic density”, “density estimation”, “density assessment”, “machine learning”, and “deep learning”, among others. The following databases were accessed and explored as part of the search:

- Google Scholar;
- IEEE Explore;
- arXiv;
- Springer;
- Science Direct;
- PubMed.

A total of 27 works were selected to be included in the review. Of the 27 works, 5 cover visual methods, 3 cover density estimation software, 16 cover machine learning methods, and 3 cover segmentation-based methods.

Since this review focuses extensively on AI-based approaches to breast density estimation, very few visual or software-based methods were selected. Any visual/software methods that had not been applied or evaluated in clinical settings were excluded from the review. The age of the studies did not have any impact on the inclusion of works introducing visual/software-based methods.

For AI-based methods, the selected papers focus mainly on introducing methods for mammographic density estimation. Although many papers exist in the mammogram processing literature covering a variety of tasks, such as mammographic image improvement and breast cancer risk assessment and detection in mammograms, only papers primarily addressing the issue of breast density estimation or assessment were included in this review. Regarding machine-learning-based methods, papers published before 2010 were excluded from the review to ensure that all the approaches covered are still relevant. Specifically, for deep-learning-based approaches, the oldest selected work was published in 2018. Concerning segmentation-based approaches to breast density estimation, due to the small number of works, the oldest selected study was published in 2007. In all cases, works not published in English were excluded from this review.

## 3. Results

In this section, breast density estimation methods found in the literature are reviewed and discussed. The methods detailed can be classified into four categories: visual, software-, machine learning-, and segmentation-based methods. In this review, a special emphasis is placed on machine-learning-based approaches, given their recent emergence as a viable and successful solution for several breast imaging problems, including tumor detection and classification, mammographic image improvement, and breast cancer risk assessment [21].

### 3.1. Visual Methods

Visual methods are widely adopted for breast density estimation. They can be based on parenchymal patterns, qualitative, or semi-quantitative methods [22]. The most commonly used qualitative density categorization method is the Breast Imaging Reporting and Data System (BI-RADS), created by the American College of Radiology (ACR) [23]. It can be used to categorize breasts into one of four qualitative categories:

1. Fatty (I): nearly no fibro-glandular tissue; almost entirely composed of fat;
2. Scattered areas (II): relatively small amount of fibro-glandular tissue;
3. Heterogeneously dense (III): large amount (>50%) of fibro-glandular tissue;
4. Extremely dense (IV): almost entirely made up of fibro-glandular tissue.

Other visual methods include the Wolfe [24] and Tabár [25] classifications, both of which classify breasts into five density categories based on parenchymal patterns. A few semi-quantitative visual approaches have been proposed to obtain a numeric approximation of breast density. Norman Boyd et al. categorized breasts into six density ranges based on visual estimates [5]. The Visual Analogue Scale—a 100 mm-long scale—has also been used to quantify breast density composition based on visual estimates with high accuracy [22,26].

### 3.2. Software-Based Methods

Since visual methods are highly subjective, the use of software has been proposed as a quantitative alternative. Some density estimation software is semi-automatic, requiring input from the attending radiologists. One such software is Cumulus, which calculates the percentage of radiographically dense tissue within a breast based on a radiologist’s annotation of dense region edges within an input mammogram [27]. Several studies have affirmed the effectiveness of Cumulus and the reproducibility of its estimates [22]. Other software is fully automatic, requiring little to no effort from radiologists. Such software is meant to be easily integrable and can either be area-based or volume-based. Area-based methods rely on two-dimensional area measures of breast density. A notable system for area-based assessment is Densitas, which employs two deep learning models: one to estimate percent density and another to produce a descriptive classification aligned with the four BI-RADS classes [22]. Densitas is currently cleared for clinical use in the United States, Europe, Canada, and Australia. Volume-based methods estimate the physical volume of dense tissue within the breast. Quantra [28] and Volpara [29] are two systems that fall into this category. They estimate breast volume, segment it based on density, and calculate the percentage of dense tissue. While both systems operate similarly, they differ in their physics models, with Volpara using a relative physics model and Quantra using an absolute physics model. Both Quantra and Volpara have received clearance from the United States Food and Drug Administration (FDA).

### 3.3. Machine-Learning-Based Methods

**Figure 2:**
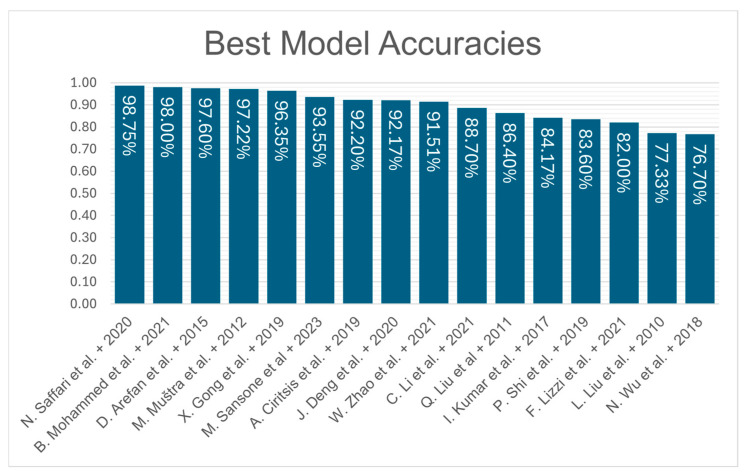
Summary of best accuracies of models proposed in reviewed works [30,32,33,34,35,36,37,38,39,40,41,42,43,44,45,49].

Several works in the literature use machine learning for breast density estimation. These works introduce methods for mammogram preprocessing and feature extraction to facilitate notable estimation performance. The methods employed can be broken down into two categories: traditional machine learning and deep learning approaches. Most machine learning methods in the literature produce qualitative estimates in accordance with the BI-RADS classification scheme. A summary of the best accuracies achieved by the reviewed works is showcased in Figure 2.

In terms of image preprocessing, various procedures are covered by different works. Region of interest (ROI) extraction is a common element among the majority of works in the literature, since works that extract a ROI tend to perform best [30]. Further, the work in [31] suggests that the central region of the breast is the most indicative of differences between density categories. The works in [30,32,33] detail methods for artifact and in-image label removal, as well as pectoral muscle removal. Lastly, the works in [34,35,36,37,38,39,40] mention rescaling input mammograms to speed up the subsequent steps.

Traditional machine learning methods generally rely on hand-crafted feature extraction procedures, where statistical and/or textural features are extracted. Statistical feature extraction involves computing the statistical properties of input data. In terms of mammograms, this can refer to features such as the mean luminance, the standard deviation, the skewness and the kurtosis. Statistical feature extraction is undertaken in [30,32,33,41,42,43]. Textural feature extraction is commonly performed using a gray-level co-occurrence matrix (GLCM). GLCM is a statistical method that reflects the texture of an image by calculating the co-occurrences of particular pixel gray-level values at a specified spatial resolution (i.e., distance and direction). As demonstrated in [32,41,42], GLCM can produce diverse amounts of features depending on the input parameters. Statistical methods can also be used to extract shape descriptors and describe features such as the symmetry of fibro-glandular tissue in the breast [32].

In contrast, deep learning methods can learn complex features from input mammograms independently. Those features can represent texture, scale, and orientation, among other details that are difficult to represent otherwise [34]. The most commonly used type of neural network for breast density estimation is the convolutional neural network (CNN), as seen in [34,35,36,37,38,39,40,44,45].

With respect to the evaluation metrics, traditional classification metrics are utilized in all referenced works, as summarized in Table 1. The main metrics seen are classification accuracy and F1-score. Some works specify AUC, precision, recall, specificity, sensitivity, and loss.

With regard to data, three publicly available datasets are utilized by several works in the literature. Specifically, the public datasets used are Mini-MIAS [46], DDSM [47], and INbreast [48]. A variety of private datasets are also employed in the literature. Table 2 details the public and private datasets used in the breast density estimation literature.

**Table 2:** Datasets utilized in the literature.

Dataset	Availability	Origin	Views	Total Images	Density Categories
Mini-MIAS [46]	Public	UK	MLO	322	3
DDSM [47]	Public	USA	CC, MLO	10,480	4
INbreast [48]	Public	Portugal	CC, MLO	410	4
Tianjin Tumor Hospital [43]	Private	China	CC, MLO	88	4
University Hospital Zagreb [42]	Private	Croatia	MLO	144	2, 3, and 4
Gansu Provincial Cancer Hospital [30]	Private	China	MLO	128	3
University Hospital “Luigi Vanvitelli” [49]	Private	Italy	CC, MLO	876	4
New York University School of Medicine [45,50]	Private	USA	CC, MLO	886,000	4
University Hospital Zurich [35]	Private	Switzerland	CC, MLO	20,578	4
First Hospital of Shanxi Medical University [44]	Private	China	CC, MLO	18,157	4
University Hospital of Pisa [39,51]	Private	Italy	CC, MLO	6648	4
Retrospective Study [40]	Private	China	CC, MLO	1985	4

#### 3.3.1. Traditional Machine Learning Approaches

As previously mentioned, traditional methods rely on actively selected and extracted features. Those features can be textural, statistical, or, as is most often the case in breast density assessment, a combination of both. The reviewed traditional machine learning approaches are highlighted in Table 3.

In ref. [30], GLCM was used at all pixel distances in all directions to create a feature vector of 528 dimensions. A total of 24 matrices were used to produce 22 features each. Additionally, the mean, skewness, and kurtosis are extracted for each mammographic image. Interestingly, ref. [30] also computed a density ratio based on pixel sums and combined it with features derived statistically and through GLCM, resulting in a 532-dimensional feature vector. This vector was then used to train a one-against-one SVM model as the primary model, as well as an extreme learning machine (ELM) model—a type of feed-forward neural network detailed in [52]—for comparison. The models in [30] were trained and tested on three datasets—mini-MIAS, DDSM, and a combination of mini-MIAS and a private dataset—and validated using 10-fold cross-validation. The best performing model was a linear kernel SVM, with the ELM model fading in comparison. The insights gained highlight the effectiveness of GLCM for texture analysis, the value of combining statistical data with density ratios, and the robustness of linear SVMs for high-dimensional data.

In ref. [32], nine statistical features, including the mean, standard deviation, and smoothness, are extracted from the histogram of each ROI. Those features were then fed into a two-layer feed-forward neural network with a sigmoid function. The model was trained and tested over five iterations using the mini-MIAS database, and the accuracies were reported for varied numbers of hidden layers. The highest average accuracy was achieved by the model with eight hidden layers. In contrast, the worst-performing model was the one with only two hidden layers. Comparatively, every other model performed well. However, it is worth noting that only 43 images were used in the training and testing of the algorithm. Nevertheless, the findings suggest that notable performance can be achieved with as little as four hidden layers and using a relatively small feature vector.

The authors of [33] calculated six statistical features—composed mainly of variance, skewness, and kurtosis, as well as mean values—at three histogram resolutions, to come up with an 18-dimensional feature vector. The features were then fed into a Directed Acrylic Graph SVM (DAG-SVM)—a one-against-all SVM. The model was tested on the MIAS database and validated using leave-one-out cross-validation. The model achieved modest accuracy, performing best in classifying fatty breasts and worst in classifying glandular breasts. This discrepancy highlights a difficulty in distinguishing the subtler features of dense tissue, indicating potential areas of improvement in feature extraction and selection.

In ref. [41], eleven first-order statistical features—including energy, uniformity, and entropy—are computed from ROI histograms. Additionally, 13 GLCM features, 5 gray-level difference statistics (GLDS) features, 11 gray-level run length matrix (GLRLM) features, and 210 Law’s texture energy features are extracted. Furthermore, ref. [41] employs the 2D Gabor Wavelet Transform (2D GWT) to generate features that capture Gabor-filtered statistical details, including the mean and standard deviation. This process is performed for 21 images, producing 42 features. All extracted features are then input into six hierarchical models with different architectures. The models used in [41] aim to decompose the classification task from a four-class problem into three binary classification problems. Specifically, the first stage of each model separates BI-RADS I breasts from the rest, the second stage differentiates BI-RADS II breasts, and the third stage classifies breasts as either BI-RADS III or BI-RADS IV. In implementing this architecture, KNN, probabilistic neural network (PNN), artificial neural network (ANN), neuro fuzzy classifier (NFC), and SVMs, were utilized. In all but the sixth model, a hierarchy of three blocks of the algorithm was implemented, each preceded by Principal Component Analysis (PCA) for dimensionality reduction. The sixth model followed the same hierarchy but incorporated the three best-performing algorithms (one for each step): SVM, NFC, and KNN. All models were trained and tested using the DDSM database. Among the first five models, PCA-NFC achieved the best performance, followed by PCA-SVM and PCA-KNN. The hybrid model, however, outperformed all the others, achieving notable classification accuracy. The results of this work highlight the potential of ensemble models, and indicate that certain models can better distinguish between specific BI-RADS classes than others—i.e., SVM was best for identifying BI-RADS I breasts, NFC was best for recognizing BI-RADS II breasts, and KNN was best for separating BI-RADS III and BI-RADS IV breasts. The hybrid model effectively utilizes each individual model to attain promising results.

The work in [42] implemented GLCM at 4 directions and 4 distance values to extract 228 features. The authors also extracted several different features from 16-, 32-, and 256-bin histograms. Two variations of KNN (1-NN and 5-NN) and a Naïve Bayesian model were deployed. The models were tested on the mini-MIAS dataset, as well as on a private dataset, and were validated using leave-one-out cross-validation. The models were also tested on different variations in the number of categories; the models were used to classify mammograms into two, three, and four categories in different iterations and using variations in the datasets. The best-performing model for both datasets was 1-NN, with much higher accuracy in binary classification than in multiclass assessment, and better performance on the private dataset than on the mini-MIAS dataset. The authors of the study attributed the variation in performance to the higher image quality in the private dataset.

In ref. [43], mammographic images were divided into subregions, and histogram moments were used to generate features that measure the variance, skewness, kurtosis, and means of each subregion. These features were then input into an SVM, which was employed to classify the subregions into high-density and low-density categories. Based on the SVM output, the density was estimated as the ratio of high-density subregions to the total number of subregions. This numerical density estimate was subsequently used to classify the mammogram into one of four density categories. The model was trained and tested on a private dataset, achieving significant accuracy. It correctly identified all of the class I and class IV breasts, but struggled with class II and class III breasts. This indicates that breasts on the extremities (BI-RADS I and BI-RADS IV) have more distinctive features that make them more readily distinguishable. The difficulty in capturing subtle features that could help identify BI-RADS II and BI-RADS III breasts remains.

The authors of [49] designed an elaborate feature extraction procedure and tested multiple models. First, they applied histogram equalization to mammograms to enhance image quality. They also removed the pectoral muscles and isolated the breast tissue. Next, they extracted 112 GLCM features, 45 Law’s texture energy features, 28 GLRLM features, 30 discrete wavelet transform (DWT) features, 12 histogram features, fractal dimensions, and local binary patterns. For feature selection, filter feature selection through correlation and wrapper feature selection through Recursive Feature Elimination (RFE) were compared. The extracted features were used to train four separate models: SVM, Linear Discrimination Analysis (LDA), ANN, Decision Tree (DT), and Random Forest Tree. The authors transformed the problem into a binary classification task by merging BI-RADS I and II cases into a “fatty” class and BI-RADS III and IV cases into a “dense” class. For evaluation, 10-fold cross-validation was utilized. The best performance was achieved using the SVM model, suggesting that a comprehensive feature extraction procedure can lead to significant breast density classification accuracy.

#### 3.3.2. Deep Learning Approaches

Deep learning approaches do not require hand-crafted features. Instead, neural networks learn complex features from input mammograms, and can reliably be used to estimate breast density. Table 4 summarizes the reviewed deep-learning-based breast density estimation approaches.

Uniquely, in ref. [34], binary masks of input mammograms were generated using a conditional generative adversarial network (cGAN). Features of input mammograms are learned by the encoder of the cGAN’s generator, while binary masks are produced by the decoder of the generator. The masks are subsequently fed to a CNN consisting of three convolutional layers and two fully connected layers. The CNN classifies an input mammogram into one of four density categories. The model also generates a numerical density estimate based on a quotient of the number of dense tissue pixels and the number of all breast tissue pixels. The INbreast database was used to train and evaluate the model, utilizing balanced and imbalanced input data of different sizes (128 × 128 and 64 × 64 pixels). The best performance was attained using a balanced dataset with a standard input size of 128 × 128 pixels. For the imbalanced dataset, the model achieved better results with an input size of 64 × 64 pixels. The study’s findings suggest that the combination of cGAN and CNN is effective for breast density classification.

The study in [35] employed a DCNN with thirteen convolutional layers, four dense layers, and a fully connected softmax classification layer. The model was trained and tested using MLO and CC mammograms from a private database. The model achieved significant accuracy for both, but showcased better performance in the classification of MLO mammograms. The findings suggest that DCNNs can provide standardized and observer-independent breast density classifications, potentially improving clinical accuracy.

In ref. [36], a lightweight CNN architecture was used to extract deep features from mammographic images. The CNN comprises three convolutional layers (with max pooling implemented) and three fully connected layers. The model was trained and tested on the mini-MIAS database and validated using five-fold cross-validation. To address the limited number of images in the mini-MIAS dataset, image augmentation was applied. The authors also explored various segmentation methods (i.e., preprocessing) and experimented with different numbers of convolutional layers. The primary three-layer model achieved the best reported accuracy. Regarding segmentation, the best-performing model was the one that extracted pectoral muscles alongside the breast region. The authors conclude that utilizing a balanced CNN architecture along with image augmentation can result in improved breast density classification accuracy.

In ref. [37], a transfer learning approach was implemented. A pretrained Inception_ResNet_V2 was implemented, within which the top layer was replaced by a pooling layer, followed by two fully connected layers, a dropout layer, and a softmax classification layer. The model was trained on the INbreast database and had significant classification accuracy. This work demonstrated the effectiveness of transfer learning in achieving high performance, even with a small training dataset.

Another study, ref. [38], introduced an architecture named BASCNet, which integrated ResNets from [50] with an adaptive spatial attention module (ASAM) and an adaptive channel attention module (ACAM). In the model described in [38], ASAM is utilized to capture distinctive features, while ACAM highlights informative channels. The model was trained on CC mammograms from the DDSM database as well as on MLO and CC mammograms from the INbreast database. The model showcased robust performance overall, with better classification accuracy observed on mammograms from the INbreast database. The authors concluded that the addition of the attention modules allowed the model to capture distinctive spatial and channel dimension features, consequently resulting in robust performance.

The authors of [39] used a very deep residual CNN. The CNN consists of 41 convolutional layers arranged in residual blocks. The model was trained and tested on a private dataset using two variations of input images: images with and images without pectoral muscles. The model performed better on mammograms where the pectoral muscle had been removed, with the accuracy dropping slightly for images where it had been kept. This finding underscores the importance of mammogram preprocessing in improving density assessment performance.

The approach detailed in [40] made use of ResNet, which was originally proposed in [53]. In ref. [40], the authors added several dilated convolutional and channel-wise attention layers to the architecture. The model was trained and tested on a private dataset as well as on the INbreast database. The proposed model exhibited notable performance on the private dataset, but was less impressive when applied to the INbreast database. This discrepancy highlights the challenges of generalizing across different datasets. The authors suggest fine-tuning as a viable solution, since it requires fewer samples and speeds up the training process compared to training from scratch.

The work in [44] improved on the basic CNN structure by introducing squeeze-and-excitation (SE)-Attention blocks to the architecture. Three different CNN models—Inception_V4, ResNeXt, and DenseNet—were augmented with SE-Attention blocks, consequently tested on a private database, and validated using 10-fold cross-validation. The best performing model was the augmented Inception_V4 model, with strong classification accuracy. The authors of this work concluded that the SE-Attention mechanism can significantly enhance the feature extraction ability and the overall performance of CNN models for density estimation.

In ref. [45], transfer learning was applied with a deep CNN (DCNN) that was previously trained and used in [50] for cancer screening. The architecture in [45] was identical to that in [50], with the exception of a softmax classification layer. This model was then tested on a massive, private dataset. The authors of [45] experimented with different input data amounts, ranging from 1% of the data to 100% of the data. In all cases, the model showcased modest top-1 accuracies, significant top-2 accuracies, and notable superclass (dense vs. not dense) accuracies. Performance only improved marginally with the addition of more input data, suggesting that the volume of data may not be the most significant factor in improving breast density estimation accuracy.

### 3.4. Segmentation-Based Methods

The machine learning methods described earlier are supervised, relying on subjective expert assessment for their training. Mammogram segmentation is a mainly unsupervised alternative that can be used to estimate breast density. Segmentation of the breast region can be performed to gain a quantitative measure of overall breast density and can often be used for cancer risk assessment in mammograms [17]. Automatic density segmentation methods can be arranged into two categories: area density projection-based and volume density projection-based methods. Area density projection-based methods include thresholding, clustering, statistical modeling, and collective multiple measurement approaches. Volume density projection-based methods estimate the depth of a given image to compute volume and consequently use it to segment the image. These include prior calibration, in-image reference calibration, and software solutions such as Quantra (version 1.2β) [28] and Volpara (Version 1.2.1) [29].

Segmentation methods have been applied directly to breast density assessment to produce continuous percent density estimates by following the segmentation process with arithmetic division. A summary of the detailed approaches is provided in Table 5.

The work in [54] presents an unsupervised approach to the estimation of mammographic percent density. Multiple segmentation steps are applied to allow for the estimation of breast density. First, a Gaussian mixture modeling approach is applied to separate the breast tissue from non-breast tissue. Next, the breast outline is automatically selected using a tracing tool, and irrelevant radiographic markers are removed. Then, K-means clustering is employed to segment the chest wall from the breast. K-means is then also applied to the breast tissue in order to segment it into an adjustable number of clusters—typically set to four to match the BI-RADS density classification scheme. Percent density is then estimated through arithmetic division. To evaluate this method, the authors computed the correlation between the resulting estimates and the qualitative BI-RADS labels. This method achieved a strong correlation for both CC and MLO mammograms. The authors conclude that the proposed approach presents a quantitative—and more objective—assessment method for breast density estimation, which may improve risk assessment.

The work in [4] combines unsupervised feature learning with supervised classification in order to estimate percent mammographic density and perform cancer risk assessment. Their method utilizes a convolutional sparse autoencoder (CSAE) that autonomously learns informative features from unlabeled mammographic images, followed by a supervised classifier (softmax regression) that estimates the probability that each pixel in a mammogram belongs to the dense class. Thresholding the output of the model results in a segmented mask of breast tissue. Percent density is then computed as the percentage of dense tissue in that mask. This approach results in a significant correlation between the automated density scores and manually assessed ones, with better performance on fatty tissues than dense tissues. Its notable performance can be attributed to the models ability to learn discriminative features through unsupervised pre-training with a CSAE, enabling effective segmentation without relying on prior assumptions.

The work in [55] presented a supervised method for the simultaneous segmentation of breast areas and dense tissue, as well as the calculation of percentage breast density via arithmetic division. The method involved a multitask deep learning model (MTLSegNet) that utilizes multilevel dilated residual blocks and parallel dilated convolutions to enhance feature extraction. The model is trained using expert-annotated segmentation masks of mammograms from three datasets—mini-MIAS, DDSM, and a private dataset. The percentage density is calculated by comparing the area of dense tissue to the total breast area. The study’s segmentation performance was evaluated using the F-score and intersection over union (IoU) metrics. The study’s results demonstrated that MTLSegNet outperformed baseline models, showing higher F-scores and IoU metrics. Furthermore, the percentage density estimates correlated very strongly with radiologists’ assessments. This work demonstrates that utilizing a diverse dataset, with mammograms from different sources with varying resolutions and intensities, can improve the generalizability of supervised models.

## 4. Limitations and Future Directions

The breast density estimation methods detailed in Section 3 are varied, and each has advantages and disadvantages. In this section, a discussion is provided, wherein the issues of each reviewed approach are summarized, and future research directions are outlined.

Visual methods—specifically the BI-RADS system—are the most widely adopted standard for breast density estimation. However, they tend to be rather subjective, and their efficacy is proportional to the experience of the attending radiologist. Additionally, they are labor-intensive and relatively primitive, especially when compared to other more precise methods of measurement. In spite of that, visual methods remain the most commonly adopted methods, particularly due to them not requiring any new infrastructure to implement them. This cost-efficiency, coupled with the widespread familiarity of radiologists with the BI-RADS system, make visual methods very practical, especially in smaller clinical environments.

Breast density estimation software presents a viable solution to the problems with visual estimation. Nevertheless, the available software solutions each have their drawbacks. Semi-automatic software solutions—chief among which is Cumulus—still require the input of an expert. Fully automatic systems, such as Densitas, Quantra, and Volpara, are more convenient than their semi-automatic counterparts, but they have their own issues. They are entirely proprietary, and not much is known about the exact methods of their functionality. This matter introduces issues such as interoperability difficulties, data ownership concerns, and limited advancement in quality. It also makes them somewhat difficult to accurately assess, and limits any collaborative innovation efforts. Some of this software employs machine learning techniques, which means that their algorithms are likely trained on expert labels, possibly making their assessments subjective. They may also have trouble generalizing to different demographics—they are cleared in varying markets, and as such, their algorithms may be trained and tuned for those specific markets. Furthermore, their estimates vary, and can be fairly inconsistent. For example, Volpara may underestimate, while Quantra may overestimate mammographic density [56]. It is also worth noting that all software solutions require the deployment of infrastructure that can support their operation, introducing set-up and maintenance costs. Furthermore, radiologists need to be trained on the specific software utilized in their clinics, and may have trouble migrating from one software to another after years of experience. All of these issues discredit the practicability of breast density estimation software in clinical settings. Nonetheless, in contrast with AI-based methods, breast density estimation softwares have been extensively studied and evaluated in clinical environments, with notable success as support tools for radiologists.

The machine learning methods in the literature, both the traditional and deep learning ones, are promising. A notable classification performance has been achieved on all publicly available datasets as well as on several private datasets [30,32,34,35,37,38]. However, the machine learning methods discussed in Section 3.3 all rely on labeled data to act as ground truth. This is problematic because, as detailed earlier, expert labels can vary significantly and be considerably subjective. Additionally, for all but one study [34], the machine learning literature focused mainly on classification according to BI-RADS, and not on the quantitative estimation of percentage breast density—which can be more precise and informative.

Segmentation-based approaches provide a solution that can compute quantitative breast density estimates, but they are not without issues. The segmentation-based methods reviewed in Section 3.4 involve some innovative approaches, but outside of the work in [54], they are supervised, requiring expert-annotated breast density segmentations for training. On the other hand, even though a deliberate procedure was described in [54], the method employed—K-means—is relatively primitive, making use of suboptimal techniques.

In terms of applicability, generalizability is a major concern for AI-based models, as they often perform well in the conditions they were trained in, but may struggle to adapt to diverse patient populations or different clinical environments. Both machine-learning- and segmentation-based approaches may require expensive hardware to run. This is especially true for methods relying on deep learning, which may require powerful GPUs to run efficiently. The physical hardware requirement can be bypassed by using the cloud for storage and processing, but this might introduce new costs and data privacy issues. Additionally, the collection of the high-quality, labeled data needed for model training can be highly costly, further reducing the practicality of supervised approaches. However, these issues are all addressable. One way to address generalizability is by developing models that are trained on diverse datasets representing a broad range of populations and clinical scenarios. This can be achieved through international collaboration, and can improve the adaptability of supervised models to different populations. Furthermore, effective lightweight models, such the one proposed in [36], exist in the literature. Such models can be deployed on consumer-grade devices, making them more practical than software solutions. It is also important to point out that unsupervised approaches inherently address the issues of subjectivity and generalizability, since they do not rely on labeled data, and are not constrained by a specific training dataset. Thus, an unsupervised approach utilizing a lightweight architecture may be ideal for a clinical environment.

When AI is discussed in the context of clinical implementation, a few ethical concerns arise. First, the issue of data privacy must be addressed. The data collected from patients must be anonymized, and any personal health data must be kept confidential. In publicly available datasets, personal details such as the patient’s age are often omitted in compliance with confidentiality concerns. Additionally, informed consent should be obtained from patients in order to comply with ethical standards. Approval from ethical boards of institutions is generally required before data can be collected. Furthermore, it is paramount that AI models adhere to data protection laws and regulations, which can vary by region. The issue of data privacy often complicates the data collection process, but it serves to protect the privacy of patients. Second, the implications of AI misclassification must be considered. Misclassification can lead to patient harm; a false negative can get in the way of timely intervention, while a false positive may lead to unnecessary treatments, tests, or procedures. Notably, in the context of breast density, overestimating a patient’s density may spur the attending radiologist to request more invasive procedures, while underestimating density may delay cancer diagnosis, or worse, prevent it entirely. In addition, an AI model that fails frequently will lose the trust of patients and professionals. If clinicians do not trust AI tools, they would be reluctant to integrate them into their practices. Third, it is important to consider the transparency and explainability of AI algorithms. AI models often function in a way that is difficult to understand, and this can make patients uneasy. They also raise issues of accountability—clear lines on which are necessary to ensure that responsibility for patient outcomes remains with healthcare institutions. Last, the overuse of AI in healthcare could serve to dehumanize it. It is crucial that AI-based healthcare tools—including breast density estimation systems—aim to complement human decision-making, not replace it. Clinicians should retain the ability to override AI recommendations when necessary.

Future research into automated breast density estimation must address these limitations; namely, it should offer solutions to subjectivity and cost-inefficiency, which make the current approaches impracticable in a real-world clinical setting. To that end, future work should involve solutions that rely less on expert labels, given the high inter-observer variability among radiologists. One possibility might be to utilize unsupervised learning, as it deals with unlabeled data, and can be used to facilitate quantitative density estimates through image segmentation. Unsupervised segmentation techniques include active contour models, which have been successfully implemented for breast cancer detection [57]. Future work should also focus on employing state-of-the-art deep-learning models, as they tend to be more effective than their traditional counterparts. The recent success of transformer models in medical imaging tasks, including breast tumor segmentation and cancer detection, makes them a potentially suitable option for breast density estimation [58]. A notable transformer-based approach can be seen in [59], where an encoder–decoder architecture with integrated transformer and ResNet modules is utilized for pixel-level segmentation of mammogram ROIs. In addition, the impact of image enhancement techniques on breast density estimation models is not well studied. Techniques such as contrast-limited adaptive histogram equalization (CLAHE) and super-resolution should be explored in the context of breast density estimation, given their proven ability to significantly improve the image quality of mammograms [60,61]. Furthermore, augmentation-reliant approaches should be forgone in favor of approaches that promote generalizability. Lastly, proposed solutions should be tested on more than one dataset in order to obtain a reliable measure of performance. By addressing these aspects, future research can pave the way for less subjective, more cost-efficient, and practical methods for breast density estimation, ultimately improving clinical outcomes and patient care.

## 5. Conclusions

Breast density is an important risk factor for breast cancer and inversely related to the sensitivity of screening mammography. In this work, a review of the literature surrounding breast density estimation, focusing primarily on methods utilizing machine learning, was presented. The review highlighted significant advancements and detailed approaches proposed in the literature. The limitations of current approaches—namely subjectivity, cost-inefficiency, and impracticability—were discussed, and future research directions were identified. By addressing said limitations, future research can lead to more objective, cost-effective, and practical methods for breast density estimation.

## Figures and Tables

**Table 1:** Evaluation metrics utilized in the literature.

Metric	Formula
Accuracy	$\frac{True\;Positives+True\;Negatives}{True\;Positives+False\;Positives+True\;Negatives+False\;Negatives}$
F1-Score	$\frac{2\times Precision\times Recall}{Precision+Recall}$
Precision	$\frac{True\;Positives}{True\;Positives+False\;Positives}$
Recall	$\frac{True\;Positives}{True\;Positives+True\;Negatives}$
Specificity	$\frac{True\;Negatives}{False\;Positives+True\;Negatives}$
Sensitivity	$\frac{True\;Positives}{True\;Positives+False\;Negatives}$

**Table 3:** Summary of traditional machine learning approaches to breast density estimation.

Study	Dataset	Preprocessing	Feature Extraction	Model(s)	Results (Accuracy)
L. Liu et al., 2010, [33]	MIAS	Removal of labels and pectoral muscle	Statistical feature extraction	DAG-SVM	77.33%
Q. Liu et al., 2011, [43]	Private database	Removal of artifacts, pectoral muscle, and contour line, as well as enhancement of images through dyadic wavelet transform	Statistical feature extraction	SVM	86.40%
M. Muštra et al., 2012, [42]	Mini-MIAS and a private database	Removal of artifacts, reorientation and resizing of the breast, and extraction of the ROI	Statistical feature extraction and GLCM	KNN (k = 1 and k = 5) and Naïve Bayes	MIAS: 1-NN: 90.37% 5-NN: 89.44% Naïve Bayes: 91.61% Private database: 1-NN: 97.22% 5-NN: 90.28% Naïve Bayes: 89.58%
D. Arefan et al., 2015, [32]	Mini-MIAS	Denoising and removal of artifacts and pectoral muscle	Statistical feature extraction	Neural network with sigmoid function	97.6%
I. Kumar et al., 2017, [41]	DDSM	Extraction of ROI from central breast region	Statistical feature extraction, GLDS, GLCM, GLRLM, Law’s texture energy, and 2D GWT	PCA-KNN, PCA-PNN, PCA-ANN, PCA-NFC, PCA-SVM, and a Hybrid Hierarchical Framework	PCA-KNN: 72.50% PCA-PNN: 68.33% PCA-ANN: 50.41% PCA-NFC: 80.41% PCA-SVM: 78.33% Hybrid: 84.17%
X. Gong et al., 2019, [30]	Mini-MIAS, DDSM, and a private database	Denoising and removal of labels and pectoral muscle	Statistical feature extraction, GLCM, and area-based density estimation	SVM	MIAS: 96.19% DDSM: 96.35% MIAS and private database (mixed): 95.01%
M. Sansone et al., 2023, [49]	Private database	Histogram equalization and pectoral muscle removal	Statistical feature extraction, GLCM, Law’s texture energy, GLRLM, and DWT	SVM	93.55%

**Table 4:** Summary of deep learning approaches to breast density estimation.

Study	Datasets	Preprocessing	Model(s)	Results
N. Wu et al., 2018, [45]	Private database	-	DCNN	Accuracy: 76.70% macAUC: 0.916
A. Ciritsis et al., 2019, [35]	Private database	Rescaling	DCNN	MLO Accuracy: 92.20% MLO AUC: 0.980 CC Accuracy: 87.40% CC MLO: 0.970
P. Shi et al., 2019, [36]	Mini-MIAS	Rescaling and pectoral muscle removal	CNN	Accuracy: 83.6% Loss: 0.52
N. Saffari et al., 2020, [34]	INbreast	Removal of pectoral muscle and rescaling	cGAN-CNN	Accuracy: 98.75% Precision: 97.50% Sensitivity: 97.50% Specificity: 99.16%
J. Deng et al., 2020, [44]	Private database	Removal of pectoral muscle, grayscale transformation, cropping of images, and image whitening	CNN with SE-Attention	Accuracy: 92.17% F1-score: 90.33%
B. Mohammed and B. Nadjia, 2021, [37]	INbreast	Reorientation to left side, breast area extraction, and rescaling	Inception_ResNet_V2	Accuracy: 98% Precision: 97% Recall: 96% Specificity: 98% F1-score: 96%
W. Zhao et al., 2021, [38]	DDSM and INbreast	Denoising, breast area extraction, and rescaling	BASCNet: ResNet and ASAM and ACAM	DDSM (CC): Accuracy: 85.10% ± 2.50% F1-score: 73.92% ± 3.82% AUC: 91.54% ± 0.88% INbreast (CC and MLO): Accuracy: 90.51% ± 5.08% F1-score: 78.11% ± 10.30% AUC: 99.09% ± 1.20%
F. Lizzi et al., 2021, [39]	Private database	Conversion from 12 bits to 8 bits, rescaling, and pectoral muscle removal	Very deep residual CNN	Accuracy: 82.0% Precision: 83.3% Recall: 80.3%
C. Li et al., 2021, [40]	INbreast and a private database	Rescaling	ResNet with dilated convolutions and channel-wise attention layers	INbreast: Accuracy: 70.0% F1-score: 63.5% AUC: 84.7% Private database: Accuracy: 88.7% F1-score: 87.1% AUC: 97.4%

**Table 5:** Summary of segmentation-based methods for breast density estimation.

Study	Dataset(s)	Segmentation Techniques Utilized	Results
C. Glide-Hurst et al., 2007, [54]	Private dataset	Gaussian mixture modeling and K-means.	Spearman rho: CC: 0.67 MLO: 0.71
M. Kallenberg et al., 2016, [4]	Private dataset	Convolutional sparse autoencoder for feature learning and softmax regression for pixel labeling.	Correlation (r): 0.85 Dice coefficients: Dense: 0.63 Fatty: 0.95
N. Gudhe et al., 2022, [55]	MIAS, INbreast, mini-DDSM, and a private dataset	Multitask model with encoder–decoder architecture.	Correlation (r): 0.90

## Abbreviations

The following abbreviations are used in this manuscript:

ACAM	Adaptive Channel Attention Module
ACR	American College of Radiology
AI	Artificial Intelligence
ANN	Artificial Neural Network
ASAM	Adaptive Spatial Attention Module
AUC	Area Under the Curve
BI-RADS	Breast Imaging-Reporting and Data System
CC	Craniocaudal
cGAN	Conditional Generative Adversarial Network
CNN	Convolutional Neural Network
CSAE	Convolutional Sparse Autoencoder
DAG	Directed Acrylic Graph
DCNN	Deep Convolutional Neural Network
DDSM	Digital Database for Screening Mammography
DT	Decision Tree
DWT	Discrete Wavelet Transform
ELM	Extreme Learning Machine
GLCM	Gray-Level Co-occurrence Matrix
GLDS	Gray-Level Difference Statistics
GLRLM	Gray-Level Run Length Matrix
GPU	Graphical Processing Unit
GWT	Gabor Wavelet Transform
IoU	Intersection over Union
KNN	K-Nearest Neighbor
LDA	Linear Discrimination Analysis
macAUC	Macro Area Under the Curve
MIAS	Mammographic Image Analysis Society
MLO	Mediolateral Oblique
NFC	Neuro Fuzzy Classifier
PCA	Principal Component Analysis
PNN	Probabilistic Neural Network
RFE	Recursive Feature Elimination
ROI	Region of Interest
SE	Squeeze-and-Excitation
SVM	Support Vector Machine

## References

[1] Sung H., Ferlay J., Siegel R.L., Laversanne M., Soerjomataram I., Jemal A., Bray F. (2021). Global Cancer Statistics 2020: GLOBOCAN Estimates of Incidence and Mortality Worldwide for 36 Cancers in 185 Countries. CA Cancer J. Clin..

[2] Welch H.G., Prorok P.C., O’Malley A.J., Kramer B.S. (2016). Breast-Cancer Tumor Size, Overdiagnosis, and Mammography Screening Effectiveness. N. Engl. J. Med..

[3] Lehman C.D., Arao R.F., Sprague B.L., Lee J.M., Buist D.S.M., Kerlikowske K., Henderson L.M., Onega T., Tosteson A.N.A., Rauscher G.H. (2017). National Performance Benchmarks for Modern Screening Digital Mammography: Update from the Breast Cancer Surveillance Consortium. Radiology.

[4] Kallenberg M., Petersen K., Nielsen M., Ng A.Y., Diao P., Igel C., Vachon C.M., Holland K., Winkel R.R., Karssemeijer N. (2016). Unsupervised Deep Learning Applied to Breast Density Segmentation and Mammographic Risk Scoring. IEEE Trans. Med. Imaging.

[5] Boyd N.F., Martin L.J., Bronskill M., Yaffe M.J., Duric N., Minkin S. (2010). Breast Tissue Composition and Susceptibility to Breast Cancer. J. Natl. Cancer Inst..

[6] Lamb L.R., Fonseca M.M., Verma R., Seely J.M. (2020). Missed Breast Cancer: Effects of Subconscious Bias and Lesion Characteristics. Radiographics.

[7] Wengert G.J., Helbich T.H., Leithner D., Morris E.A., Baltzer P.A.T., Pinker K. (2019). Multimodality Imaging of Breast Parenchymal Density and Correlation with Risk Assessment. Curr. Breast Cancer Rep..

[8] Redondo A., Comas M., Macià F., Ferrer F., Murta-Nascimento C., Maristany M.T., Molins E., Sala M., Castells X. (2012). Inter- and Intraradiologist Variability in the BI-RADS Assessment and Breast Density Categories for Screening Mammograms. Br. J. Radiol..

[9] Sprague B.L., Conant E.F., Onega T., Garcia M.P., Beaber E.F., Herschorn S.D., Lehman C.D., Tosteson A.N.A., Lacson R., Schnall M.D. (2016). Variation in Mammographic Breast Density Assessments among Radiologists in Clinical Practice: A Multicenter Observational Study. Ann. Intern. Med..

[10] Duijm L.E.M., Louwman M.W.J., Groenewoud J.H., van de Poll-Franse L.V., Fracheboud J., Coebergh J.W. (2009). Inter-Observer Variability in Mammography Screening and Effect of Type and Number of Readers on Screening Outcome. Br. J. Cancer.

[11] Chung Y.R., Jang M.H., Park S.Y., Gong G., Jung W.-H. (2016). Interobserver Variability of Ki-67 Measurement in Breast Cancer. J. Pathol. Transl. Med..

[12] Birdwell R.L. (2009). The Preponderance of Evidence Supports Computer-Aided Detection for Screening Mammography. Radiology.

[13] Sak M.A., Littrup P.J., Duric N., Mullooly M., Sherman M.E., Gierach G.L. (2015). Current and Future Methods for Measuring Breast Density: A Brief Comparative Review. Breast Cancer Manag..

[14] Yaffe M.J. (2008). Mammographic Density. Measurement of Mammographic Density. Breast Cancer Res..

[15] Destounis S.V., Santacroce A., Arieno A. (2020). Update on Breast Density, Risk Estimation, and Supplemental Screening. Am. J. Roentgenol..

[16] Bodewes F.T.H., van Asselt A.A., Dorrius M.D., Greuter M.J.W., de Bock G.H. (2022). Mammographic Breast Density and the Risk of Breast Cancer: A Systematic Review and Meta-Analysis. Breast.

[17] He W., Juette A., Denton E.R.E., Oliver A., Martí R., Zwiggelaar R. (2015). A Review on Automatic Mammographic Density and Parenchymal Segmentation. Int. J. Breast Cancer.

[18] Chalfant J.S., Hoyt A.C. (2022). Breast Density: Current Knowledge, Assessment Methods, and Clinical Implications. J. Breast Imaging.

[19] Edmonds C.E., O’Brien S.R., Conant E.F. (2023). Mammographic Breast Density: Current Assessment Methods, Clinical Implications, and Future Directions. Semin. Ultrasound CT MRI.

[20] Ekpo E.U., McEntee M.F. (2014). Measurement of Breast Density with Digital Breast Tomosynthesis—A Systematic Review. Br. J. Radiol..

[21] Dhou S., Alhusari K., Alkhodari M. (2024). Artificial Intelligence in Mammography: Advances and Challenges. Artificial Intelligence and Image Processing in Medical Imaging.

[22] Destounis S., Arieno A., Morgan R., Roberts C., Chan A. (2017). Qualitative Versus Quantitative Mammographic Breast Density Assessment: Applications for the US and Abroad. Diagnostics.

[23] Sickles E.A., D’Orsi C.J., Bassett L.W. (2013). ACR BI-RADS® Mammography. ACR BI-RADS® Atlas, Breast Imaging Reporting and Data System.

[24] Wolfe J.N. (1976). Breast Patterns as an Index of Risk for Developing Breast Cancer. Am. J. Roentgenol..

[25] Gram I.T., Funkhouser E., Tabár L. (1997). The Tabar Classification of Mammographic Parenchymal Patterns. Eur. J. Radiol..

[26] Astley S.M., Harkness E.F., Sergeant J.C., Warwick J., Stavrinos P., Warren R., Wilson M., Beetles U., Gadde S., Lim Y. (2018). A Comparison of Five Methods of Measuring Mammographic Density: A Case-Control Study. Breast Cancer Res..

[27] Byng J.W., Boyd N.F., Fishell E., Jong R.A., Yaffe M.J. (1994). The Quantitative Analysis of Mammographic Densities. Phys. Med. Biol..

[28] Hartman K., Highnam R., Warren R., Jackson V. (2008). Volumetric Assessment of Breast Tissue Composition from FFDM Images. Proceedings of the Lecture Notes in Computer Science (Including Subseries Lecture Notes in Artificial Intelligence and Lecture Notes in Bioinformatics).

[29] Highnam R., Brady M., Yaffe M.J., Karssemeijer N., Harvey J. (2010). Robust Breast Composition Measurement—VolparaTM. Proceedings of the Lecture Notes in Computer Science (Including Subseries Lecture Notes in Artificial Intelligence and Lecture Notes in Bioinformatics).

[30] Gong X., Yang Z., Wang D., Qi Y., Guo Y., Ma Y. (2019). Breast Density Analysis Based on Glandular Tissue Segmentation and Mixed Feature Extraction. Multimed. Tools Appl..

[31] Li H., Giger M.L., Huo Z., Olopade O.I., Lan L., Weber B.L., Bonta I. (2004). Computerized Analysis of Mammographic Parenchymal Patterns for Assessing Breast Cancer Risk: Effect of ROI Size and Location. Med. Phys..

[32] Arefan D., Talebpour A., Ahmadinejhad N., Asl A.K. (2015). Automatic Breast Density Classification Using Neural Network. J. Instrum..

[33] Liu L., Wang J., He K. Breast Density Classification Using Histogram Moments of Multiple Resolution Mammograms. Proceedings of the 2010 3rd International Conference on Biomedical Engineering and Informatics, BMEI 2010.

[34] Saffari N., Rashwan H.A., Abdel-Nasser M., Singh V.K., Arenas M., Mangina E., Herrera B., Puig D. (2020). Fully Automated Breast Density Segmentation and Classification Using Deep Learning. Diagnostics.

[35] Ciritsis A., Rossi C., De Martini I.V., Eberhard M., Marcon M., Becker A.S., Berger N., Boss A. (2019). Determination of Mammographic Breast Density Using a Deep Convolutional Neural Network. Br. J. Radiol..

[36] Shi P., Wu C., Zhong J., Wang H. Deep Learning from Small Dataset for Bi-Rads Density Classification of Mammography Images. Proceedings of the 10th International Conference on Information Technology in Medicine and Education, ITME.

[37] Mohammed B., Nadjia B. Automated Assessment of Breast Density on Mammogram Images Based on Convolutional Neural Networks. Proceedings of the 2021 Proceedings of the International Conference on Artificial Intelligence for Cyber Security Systems and Privacy, AI-CSP 2021.

[38] Zhao W., Wang R., Qi Y., Lou M., Wang Y., Yang Y., Deng X., Ma Y. (2021). BASCNet: Bilateral Adaptive Spatial and Channel Attention Network for Breast Density Classification in the Mammogram. Biomed. Signal Process. Control.

[39] Lizzi F., Scapicchio C., Laruina F., Retico A., Fantacci M.E. (2022). Convolutional Neural Networks for Breast Density Classification: Performance and Explanation Insights. Appl. Sci..

[40] Li C., Xu J., Liu Q., Zhou Y., Mou L., Pu Z., Xia Y., Zheng H., Wang S. (2021). Multi-View Mammographic Density Classification by Dilated and Attention-Guided Residual Learning. IEEE/ACM Trans. Comput. Biol. Bioinform..

[41] Kumar I., Bhadauria H.S., Virmani J., Thakur S. (2017). A Hybrid Hierarchical Framework for Classification of Breast Density Using Digitized Film Screen Mammograms. Multimed. Tools Appl..

[42] Muštra M., Grgić M., Delač K. (2012). Breast Density Classification Using Multiple Feature Selection. Autom.-J. Control Meas. Electron. Comput. Commun..

[43] Liu Q., Liu L., Tan Y., Wang J., Ma X., Ni H. Mammogram Density Estimation Using Sub-Region Classification. Proceedings of the 2011 4th International Conference on Biomedical Engineering and Informatics, BMEI 2011.

[44] Deng J., Ma Y., Li D., Zhao J., Liu Y., Zhang H. (2020). Classification of Breast Density Categories Based on SE-Attention Neural Networks. Comput. Methods Programs Biomed..

[45] Wu N., Geras K.J., Shen Y., Su J., Kim S.G., Kim E., Wolfson S., Moy L., Cho K. Breast Density Classification with Deep Convolutional Neural Networks. Proceedings of the ICASSP, IEEE International Conference on Acoustics, Speech and Signal Processing.

[46] Suckling J., Parker J., Dance D., Astley S., Hutt I., Boggis C., Ricketts I., Stamatakis E., Cerneaz N., Kok S. (1994). The Mammographic Image Analysis Society Digital Mammogram Database. Experta Medica, International Congress Series.

[47] Heath M., Bowyer K., Kopans D., Moore R., Kegelmeyer W.P., Yaffe M.J. (2001). The Digital Database for Screening Mammography. Proceedings of the International Workshop on Digital Mammography.

[48] Moreira I.C., Amaral I., Domingues I., Cardoso A., Cardoso M.J., Cardoso J.S. (2012). INbreast: Toward a Full-Field Digital Mammographic Database. Acad. Radiol..

[49] Sansone M., Fusco R., Grassi F., Gatta G., Belfiore M.P., Angelone F., Ricciardi C., Ponsiglione A.M., Amato F., Galdiero R. (2023). Machine Learning Approaches with Textural Features to Calculate Breast Density on Mammography. Curr. Oncol..

[50] Geras K.J., Wolfson S., Shen Y., Wu N., Kim S.G., Kim E., Heacock L., Parikh U., Moy L., Cho K. (2017). High-Resolution Breast Cancer Screening with Multi-View Deep Convolutional Neural Networks. arXiv.

[51] Sottocornola C., Traino A., Barca P., Aringhieri G., Marini C., Retico A., Caramella D., Fantacci M.E. (2018). Evaluation of Dosimetric Properties in Full Field Digital Mammography (FFDM). Proceedings of the 11th International Joint Conference on Biomedical Engineering Systems and Technologies.

[52] He K., Zhang X., Ren S., Sun J. Deep Residual Learning for Image Recognition. Proceedings of the IEEE Computer Society Conference on Computer Vision and Pattern Recognition.

[53] Huang G.B., Zhu Q.Y., Siew C.K. (2006). Extreme Learning Machine: Theory and Applications. Neurocomputing.

[54] Glide-Hurst C.K., Duric N., Littrup P. (2007). A New Method for Quantitative Analysis of Mammographic Density. Med. Phys..

[55] Gudhe N.R., Behravan H., Sudah M., Okuma H., Vanninen R., Kosma V.M., Mannermaa A. (2022). Area-Based Breast Percentage Density Estimation in Mammograms Using Weight-Adaptive Multitask Learning. Sci. Rep..

[56] Rahbar K., Gubern-Merida A., Patrie J.T., Harvey J.A. (2017). Automated Volumetric Mammographic Breast Density Measurements May Underestimate Percent Breast Density for High-Density Breasts. Acad. Radiol..

[57] Malathi M., Sinthia P., Farzana F., Aloy Anuja Mary G. (2021). Breast Cancer Detection Using Active Contour and Classification by Deep Belief Network. Mater. Today Proc..

[58] Shamshad F., Khan S., Zamir S.W., Khan M.H., Hayat M., Khan F.S., Fu H. (2023). Transformers in Medical Imaging: A Survey. Med. Image Anal..

[59] Alhussen A., Anul Haq M., Ahmad Khan A., Mahendran R.K., Kadry S. (2025). XAI-RACapsNet: Relevance Aware Capsule Network-Based Breast Cancer Detection Using Mammography Images via Explainability O-Net ROI Segmentation. Expert. Syst. Appl..

[60] Umehara K., Ota J., Ishida T. (2017). Super-Resolution Imaging of Mammograms Based on the Super-Resolution Convolutional Neural Network. Open J. Med. Imaging.

[61] Ravikumar M., Rachana P.G., Shivaprasad B.J., Guru D.S. (2021). Enhancement of Mammogram Images Using CLAHE and Bilateral Filter Approaches. Cybernetics, Cognition and Machine Learning Applications: Proceedings of ICCCMLA 2020.

